# Development of Teleost Intermuscular Bones Undergoing Intramembranous Ossification Based on Histological-Transcriptomic-Proteomic Data

**DOI:** 10.3390/ijms20194698

**Published:** 2019-09-22

**Authors:** Chun-Hong Nie, Shi-Ming Wan, Yu-Long Liu, Han Liu, Wei-Min Wang, Ze-Xia Gao

**Affiliations:** 1 Key Lab of Agricultural Animal Genetics, Breeding and Reproduction of Ministry of Education/Key Lab of Freshwater Animal Breeding, Ministry of Agriculture, College of Fisheries, Huazhong Agricultural University, Wuhan 430070, China; nch44@webmail.hzau.edu.cn (C.-H.N.); wansmhzau@foxmail.com (S.-M.W.); liuhan@mail.hzau.edu.cn (H.L.); wangwm@mail.hzau.edu.cn (W.-M.W.); 2 Engineering Technology Research Center for Fish Breeding and Culture in Hubei Province, Wuhan 430070, China; 3 Department of Molecular and Cellular Biology, University of California Davis, Davis, CA 95616, USA; 4 National Demonstration Center for Experimental Aquaculture Education, Huazhong Agricultural University, Wuhan 430070, China

**Keywords:** teleost, intermuscular bone, ossification process, histological structure, genes expression

## Abstract

Intermuscular bones (IBs) specially exist in lower teleost fish and the molecular mechanism for its development remains to be clarified. In this study, different staining methods and comparative proteomics were conducted to investigate the histological structure and proteome of IB development in *Megalobrama amblycephala*, including four key IB developmental stages (S1—IBs have not emerged in the tail part; S2—several small IBs started to ossify in the tail part; S3—IBs appeared rapidly; S4—all the IBs appeared with mature morphology). Alcian blue and alizarin red S stained results indicated that IBs were gradually formed from S2 to S4, undergoing intramembranous ossification without a cartilaginous phase. A total of 3368 proteins were identified by using the isobaric tags for relative and absolute quantitation (iTRAQ) approach. Functional annotation showed that proteins which were differentially expressed among stages were involved in calcium, MAPK, Wnt, TGF-β, and osteoclast pathways which played a critical role in bone formation and differentiation. Three proteins (collagen9α1, stat1, tnc) associated with chondrocytes did not exhibit significant changes through S2 to S4; however, proteins (entpd5, casq1a, pvalb, anxa2a, anxa5) which associated with osteoblasts and bone formation and differentiation showed significantly a higher expression level from S1 to S2, as well as to S3 and S4. These further demonstrated that development of IBs did not go through a cartilaginous phase. The inhibitors of TGF-β and Wnt pathways were tested on zebrafish (sp7/eGFP) and the results indicated that both inhibitors significantly delayed IB development. This study provides a comprehensive understanding of the IB ossification pattern, which will help further elucidate the molecular mechanisms for IB development in teleosts.

## 1. Introduction

Intermuscular bones (IBs) occur only in teleosts amongst recent vertebrates, which are hard-boned spicules located in the myosepta on both sides of the vertebrae [1,2,3]. Three sets of serially homologous IBs had been reported in teleosts, which are classified referring to the site of its attachment [1,4]. Epineurals are above the horizontal septum (attach to neural arches), epipleurals are below it (attach to hemal arches or ribs), and epicentrals are in it (attach to centra). Most of the freshwater aquaculture fishes, especially Cypriniformes species, possessed a certain amount of IBs, which have an adverse effect on the edibleness and economic value of these species [3]. Since the 1960s, most studies have been focusing on IB number, morphology, and ossification process in fish species, such as in crucian carp (*Carassius auratus gibelio*), silver carp (*Hypophthalmichthys molitrix*), blunt snout bream (*Megalobrama amblycephala*), zebrafish (*Danio rerio*), Japanese eel (*Anguilla japonica*), etc. [5,6,7,8,9,10]. To date, most studies about the ossification process of IBs have remained in the morphology aspect, while details on histological structure are limited. There are two bone formation patterns that have been identified in animals. One is endochondral ossification, in which the mesenchymal cells firstly differentiated into chondrocytes and cartilage eventually replaced by bone, such as neural spines. The other type is intramembranous ossification, in which mesenchymal cells directly differentiated into osteoblast without cartilaginous phase, such as lateral halves of the clavicles and parts of the skull [11,12]. The development of IBs had been described as undergoing intramembranous ossification [12]. However, epineurals were also thought to have a cartilaginous core and cap primitively [13]. The histological or molecular analysis about IB development has not been reported to demonstrate its intramembranous ossification. Therefore, whether IB development involved a cartilage phase needs to be further investigated.

With technological development, more and more studies have focused on quantitative proteome in the post-genome era and proteomics technologies, including stable isotopes labeling by amino acids in cell culture (SILAC) [14], isotope-code affinity tags (ICAT) [15], and isobaric tags for relative and absolute quantitation (iTRAQ). Correlation analysis of proteomics and transcriptomics data contributes to multilevel regulation of gene expression processes, and it has been widely applied in many aspects. For example, Bouchal et al. [16] used transcriptome-proteomic analysis to identify potential biomarkers associated with metastatic breast cancer. Molecular mechanism of head regeneration of Hydra was analyzed and identified by transcriptome and proteome/phosphoproteome association analysis [17]. Regarding fish species, the immune response of spleen in rainbow trout (*Oncorhynchus mykiss*) at 3 days post-infection by *Aeromonas salmonicida* was investigated using Illumina-seq and iTRAQ methods [18]. The hyperosmotic-responsive genes/proteins in gills of *A. japonica* were identified with the high-throughput transcriptomic and proteomic studies [19]. Therefore, integrated analysis of histological-transcriptomic-proteomic may provide a better understanding of IB ossification pattern.

Blunt snout bream (*M. amblycephala*), belonging to Cyprinidae, is recognized as a mainly aquaculture species in Chinese freshwater fish. Our previous study revealed the molecular properties of IB development through mRNA and miRNA transcriptome analysis [20]. The current study investigated the histological structure by different staining methods (alcian blue, alizarin red S (ARS), and hematoxylin-eosin) and molecular properties of IB development through transcriptome-proteomic analysis to address the following questions: (1) whether IB ossification go through a cartilaginous phase or directly develop to bone based on histological structure with different staining methods? (2) How many proteins involved in IB ossification and which protein or protein functional groups have key functions during different IB developmental stages? The main objective of this study was to provide a comprehensive understanding of IB ossification pattern in teleosts from histological, transcriptomic, and proteomic levels. The identified candidate genes/proteins will be beneficial for understanding the molecular basis underlying the IB development in teleost.

## 2. Results

### 2.1. Histological Structures

**Figure 1:**
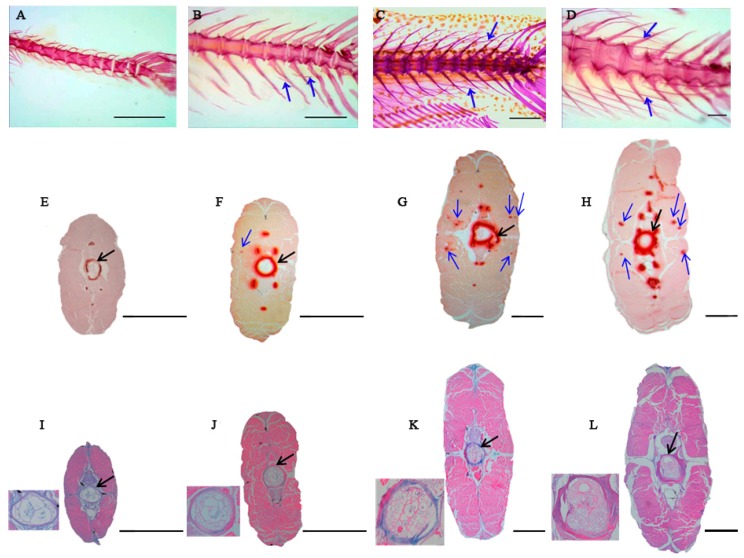
Developmental characteristics and visualization of cross sections from four key Intermuscular bone (IB) developmental stages in *M. amblycephala*. (**A**–**D**) represent ossification process of IBs stained with ARS from S1, S2, S3, and S4, respectively. (**A**) S1. Body Length ≈ 11 mm (17 dph), the IBs have not emerged in the tail part; (**B**) S2. Body Length ≈ 16mm (24 dph), several small IBs have ossified in the tail part; (**C**) S3. Body Length ≈ 21 mm (29 dph), more IBs gradually ossified in the tail part with immature form; (**D**) S4. Body Length ≈ 32 mm (42 dph), all the IBs ossified in the tail part with mature morphology. Blue arrows indicate IBs; scale bars = 200 μm. (**E**–**H**) represent histological structures of the tail part with alizarin red S staining from S1, S2, S3, and S4, respectively. (**I**–**L**) represent histological structures of the tail part with alcian blue staining from S1, S2, S3, and S4, respectively. Blue arrows indicate IBs and black arrows indicate notochord sheath. The notochord sheath and vertebra were magnified in the lower left corner in (**I**–**L**). Scale bars = 100 μm.

To understand the changes of tissue structure during the ossification process of IBs, samples from four key stages were collected from *M. amblycephala* to conduct alcian blue and ARS staining. ARS staining first appeared at IB ossification at S2 and with increasing condensations from S2 to S4 (Figure 1E–H). However, the alcian blue results showed that blue staining appeared in the notochord sheath and not in IBs’ supposed appearing part (Figure 1I–L). Moreover, hematoxylin-eosin (HE) results indicated that there were no obvious differences of muscle tissue among samples from S1 to S4 (Supplementary Figure S1), while the muscle fibers were growing a little bit.

### 2.2. General Proteome Analysis During IB Development

Proteomics data during IB development in *M. amblycephala* were obtained by iTRAQ labeling and subsequent liquid chromatography-tandem mass spectrometry (LC-MS/MS) methods. The two replicate iTRAQ samples were studied and analyzed, the coefficient of variation value indicated that the repeatability in biological replicates is quite good (Supplementary Figure S2). A total of 11,811 peptides were generated and 3688 proteins identified with at least one peptide in this study, with 3657 proteins (99.16%) being annotated with the NCBI database (Supplementary Table S1).

**Figure 2:**
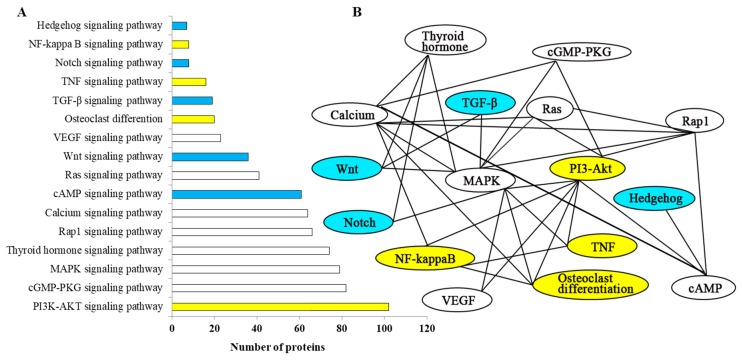
Analysis of bone-regulation pathways from identified proteins. (**A**) The number of proteins belonging to the pathways associated with IB development. (**B**) A network of pathways associated with bone. Yellow indicates pathways which are related to osteoclast, blue related to osteoblast, and white related to both osteoclast and osteoblast.

Identified proteins were functionally annotated with gene ontology (GO) and Kyoto encyclopedia of genes and genomes (KEGG), respectively. About 67.37% of the identified proteins (2269 of 3688 proteins) could be assigned to specific functions such as biological, cellular processes, and molecular functions (Supplementary Figure S3A–C). A number of proteins were functioned in more than one GO terms (Supplementary Figure S3D). Among the 3688 proteins, 3368 proteins (91.32%) were mapped into 305 KEGG pathways. Some pathways involved in bone formation were identified (Figure 2A), such as TGF-β signaling pathway, Wnt signaling pathway, osteoclasts differentiation, MAPK signaling pathway, etc. These pathways could regulate bone formation together and their relationship is shown in Figure 2B, which indicated that MAPK and calcium signaling pathways are the main pathways associated with bone development.

In order to define how many proteins regulate IB development in different stages, the proteins associated with bone development were screened out (detailed information is shown in Supplementary Table S2), such as ctsk and PGE2 associated with osteoclasts, entpd5 and smad4s related to osteoblasts, β-catenin as well as casq1a, pvalb, camk, and anxa2a/5 correlated with bone development. A relationship was constructed to understand the possible roles of these proteins during IB development (Supplementary Figure S4).

### 2.3. Comparative Analysis of Differentially Expressed Proteins in Different Comparison Groups

**Figure 3:**
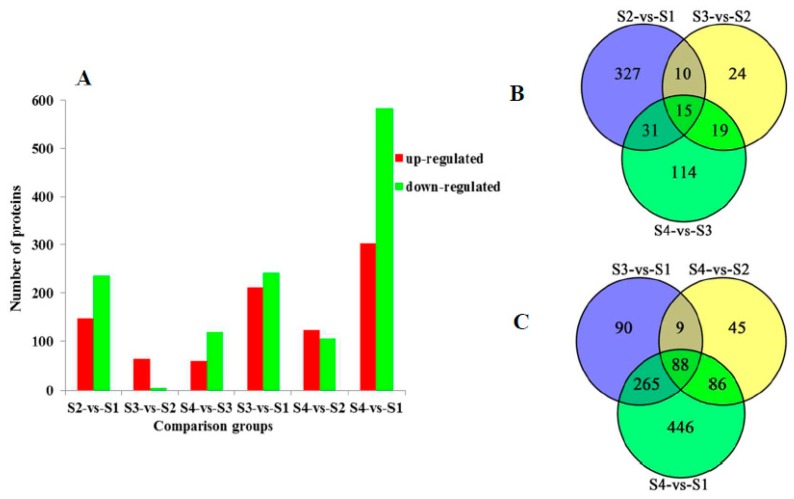
Comparison of proteins identified in six comparison groups. (**A**) indicates differential expressed proteins. X-axis: names of the comparison groups; Y-axis: the number of differentially expressed proteins; red column: up-regulated proteins; green column: down-regulated proteins. (**B**) shows a Venn diagram for differentially expressed proteins in three adjacent developmental stages. (**C**) shows a Venn diagram for differentially expressed proteins in three nonadjacent developmental stages.

To identify the differentially expressed proteins during IB development, we used a 95% confidence level (*p* < 0.05) and a cut off value of 1.5-fold for up-regulated proteins and 0.5-fold for down-regulated proteins. Comparison of adjacent developmental stages S2-vs-S1, S3-vs-S2 and S4-vs-S3 detected 147 up-regulated and 236 down-regulated proteins, 64 up-regulated and four down-regulated proteins, 60 up-regulated and 119 down-regulated proteins (Supplementary Table S3, Figure 3A) respectively, with 15 co-expressed proteins detected in all the three comparison groups (Figure 3B). Pairwise comparisons between nonadjacent developmental stages S3-vs-S1, S4-vs-S2 and S4-vs-S1 detected 211 up-regulated and 241 down-regulated proteins, 123 up-regulated and 105 down-regulated proteins, 302 up-regulated and 583 down-regulated proteins respectively (Supplementary Table S3, Figure 3A). Venn diagrams displayed 88 overlapping differentially expressed proteins were identified in nonadjacent pairwise developmental stage comparisons (Figure 3C).

**Figure 4:**
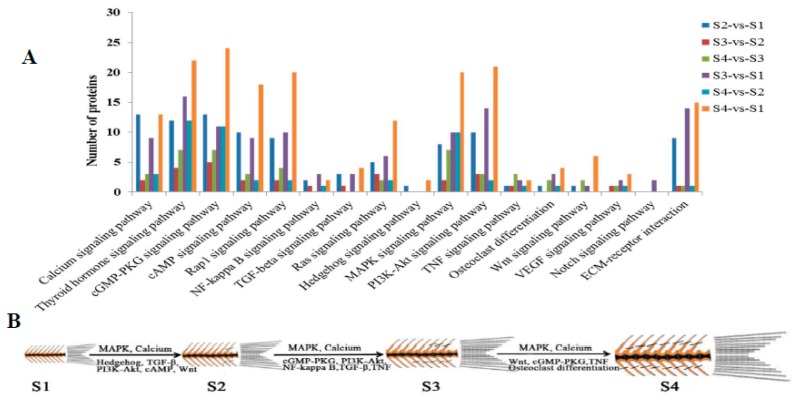
The number of bone-related pathways in six comparison groups (**A**) and the main pathways during IB developmental stages (**B**) in *M. amblycephala* based on differentially expressed proteins.

The KEGG pathway database was used to map the differentially expressed proteins and bone-regulated pathways which were found in six comparison groups, such as the MAPK pathway, PI3K-Akt pathway, NF-kappa B pathway, etc., and indicated that they may have an effect on IB development (Figure 4A). The identified bone-regulated pathways were shown in four key IB developmental stages (Figure 4B, Supplementary Table S4). The MAPK and calcium pathways were identified in S2-vs-S1, S3-vs-S2, and S4-vs-S3. Osteoclast differentiation and Wnt pathway of proteins associated with osteoclasts and osteoblasts were also identified in S2-vs-S1 and S4-vs-S3. TGF-β and NF-kappa B pathways were identified in S2-vs-S1 and S3-vs-S2, which contributes to understanding the effect on these pathways’ functions during IB development. The identification of specific expression proteins and pathways related to IB development may provide key protein information for exploring their function in this mechanism.

### 2.4. Correlation Analysis of Protein and mRNA

For better understanding of the expression trends relationship between protein expression and mRNA, integrated correlation analysis of protein and mRNA were used to investigate expression trends in this study. A total of 2804, 2801, 2773, 2801, 2801, and 2798 protein–mRNA correlation pairs were detected in six pairwise comparisons (S2-vs-S1, S3-vs-S2, S4-vs-S3, S3-vs-S1, S4-vs-S1, and S4-vs-S2) (Supplementary Table S5). The R Spearman was used to evaluate the correlation between proteomics and transcriptomics [21]. In general, the correlation level of proteomics and transcriptomics is not high (approximately 27–40%). The positive correlation was observed in all comparison groups except the S3-vs-S1 group. The R Spearman of all identified proteins for positive correlation groups including S2-vs-S1, S3-vs-S1, S4-vs-S1, S4-vs-S3, and S4-vs-S2 were 27.08%, 33.2%, 35.59%, 0.96%, 4.48%, respectively. However, the R value for the negative correlation group was −6.76% (S3-vs-S1). Meanwhile, correlation analysis of proteins was functionally classified by using KEGG pathway database. More than 88.97% of protein−mRNA correlation pairs were categorized by the KEGG database in six comparison groups. Interestingly, pathways associated with bone, according to Figure 2, were also identified, such as the calcium and MAPK pathways.

Moreover, considering this possible noise from muscle tissue, we checked the expression of genes related to muscle fibers in our transcriptomic and proteomic data. The genes of myogenic regulator factors (MRFs) family had been reported to play an important role in the regulation of the formation and differentiation of muscle fibers in animals, coding the *MyoD1*, *Myf5*, *MyoG*, *Myf6*. However, these genes were not identified in iTRAQ proteomics data, while *Myf5*, *MyoG*, and *Myf6* were identified from transcriptome data, but their expressions were not significantly changed from S1 to S4. These results indicated that the changes in musculature from S1 to S4 were not so significant (Supplementary Figure S5).

### 2.5. Network Analysis and Functional Annotation of Altered Bone-Regulated Proteins During IB Development

**Figure 5:**
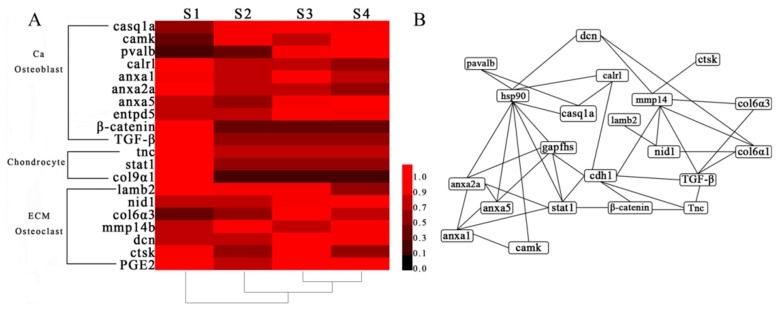
Cluster (**A**), network (**B**) and analysis of bone-regulated proteins during IB developmental stages. In the Cluster (**A**), the color intensity indicates the level of protein expression. Black indicates a low level of protein expression or undetected protein; red indicates a high level of expression.

To further explore the potential bone-related proteins in *M. amblycephala*, network interaction and functional annotation analysis were performed. A total of 20 proteins including those up- and down-regulated at protein level, and involved in bone formation and differentiation in S2-vs-S1, S3-vs-S2, and S4-vs-S3 were selected. These proteins were used to construct a protein−protein network. The result indicated that these proteins made an effect with each other on the interaction network (Figure 5A). Notably, the annotation results indicated that 17 proteins were functionally involved in bone formation processes and regulated by each other’s hsp90 and ghpdhs (glyceraldehyde-3-phosphate dehydrogenase). Besides, the altered proteins entpd5, col9α1, and prostaglandin E synthase 2 (PGE2) were not involved in the network interaction. The other four proteins were involved in network interactions and associated with bone, and all were detected during the IB development stages in *M. amblycephala*.

Based on proteomics data, 20 bone-regulated proteins were used to analyze the expression pattern based on their relative protein quantitation by cluster analysis during IB development stages and those proteins were divided into three groups (Figure 5B). The casq1a, camk, pvalb, calrl, β-catenin, TGF-β, anxa2a, anxa1, anxa5, and entpd5 represent the first group (Ca and osteoblast); col9α1, stat1, and tnc represent the second group (chondrocyte); and lamb2, nid1, mmp14, dcn, ctsk, PGE2, and col6α3 represent the third group (ECM and osteoclast). The three chondrocyte-related proteins (col9α1, tnc, stat1) had a higher expression at S1 but lower expression at S2, S3, and S4. The osteoblast-related and bone-related proteins (entpd5 and anxa1, casq1a) exhibited higher expression at S3. The calcium-related proteins (anxa5, pavlb, camk) exhibited a higher expression at S4, only one protein (mmp14) exhibited higher expression at S2.

Information of these 20 bone-related proteins is displayed in Table 1 at mRNA and protein level. In S2-vs-S1, five proteins (casq1a, camk, pvalb, entpd5, col6α3) were up-regulated at both levels and nine proteins (casq1a, camk, entpd5, pvalb, etc.) were down-regulated at both levels. Two proteins (mmp14b and nid1) were up-regulated at protein level but down-regulated at mRNA level and three proteins (anxa2a, anxa1, stat1) were down-regulated at protein level but up-regulated at mRNA level. In S3-vs-S2, two proteins (pvalb and col6α3) were up-regulated at both levels and four proteins (β-catenin, calrl, mmp14b, TGF-β) were down-regulated at both levels. Ten proteins (entpd5, anxa2a, anxa5, anxa1, casq1a, etc.) were up-regulated at protein level but down-regulated at mRNA level. In S4-vs-S3, the pvalb protein was up-regulated at both levels and five proteins (ctsk, entpd5, lamb2, PGE2, col6α3) were down-regulated at both levels. Six proteins (β-catenin, anxa5, mmp14b, camk, stat1, nid1) were up-regulated at protein level but down-regulated at mRNA level.

In summary, the different expression levels of bone-related proteins at both levels in different comparison groups may indicate that the bone-related proteins had a positive or negative effect in different IB developmental stages. Our results suggested that these 20 bone-related proteins might be involved in IB formation in *M. amblycephala* via a complex molecular regulatory mechanism.

### 2.6. Expression Validation of Bone-Related Genes

**Figure 6:**
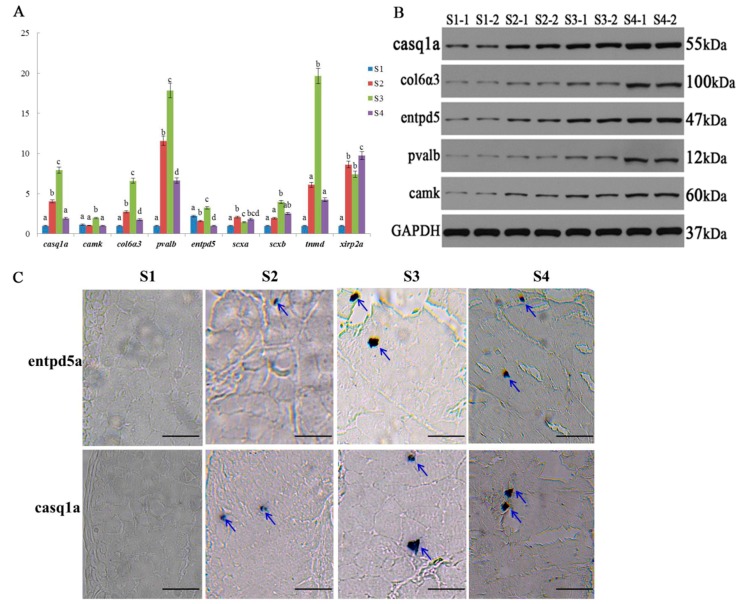
Verification of gene expression. (**A**) The relative expression of genes at different stages with qRT-PCR; for each gene, different letters above the bar of each stage indicated significant differences at the level of *p* < 0.05 among stages. (**B**) The expression levels of five proteins based on Western blot analysis; GAPDH is used as a loading control. (**C**) In situ expression analysis of *entpd5a* and *casq1a* genes at individual’s tail part from four stages. Blue arrows indicate IBs. Scale bar = 50 μm.

Overall, five genes (*entpd5*, *casq1a*, *pvalb*, *camk*, *col6α*3) associated with osteoblasts and bone formation and differentiation, and four genes (*scxa*, *scxb*, *tnmd*, *xirp2a*) related to tendon differentiation were validated with qRT-PCR and Western blot analysis. The mRNA expression of most genes showed an increasing trend from S1 to S3 and had the highest expression in S3, except *scxa* and *xirp2a* (Figure 6A). At protein level, their expression was increased during the IB developmental stages relative to the GAPDH (Figure 6B, the raw data photo of Western blot is shown in Supplementary Figure S6), which was consistent with expression data obtained from iTRAQ (Supplementary Figure S7). The two genes (*entpd5a* and *casq1a*) were further validated by in situ hybridization during the IB developmental stages (Figure 6C). These two genes showed expression at IBs in S2, S3, and S4 but not in S1. Moreover, both genes had the higher expression at S3, which was consistent with the mRNA expression results. All these validation results suggested that these genes have certain regulatory roles during IB development.

### 2.7. Pathway Inhibitor Analysis

**Figure 7:**
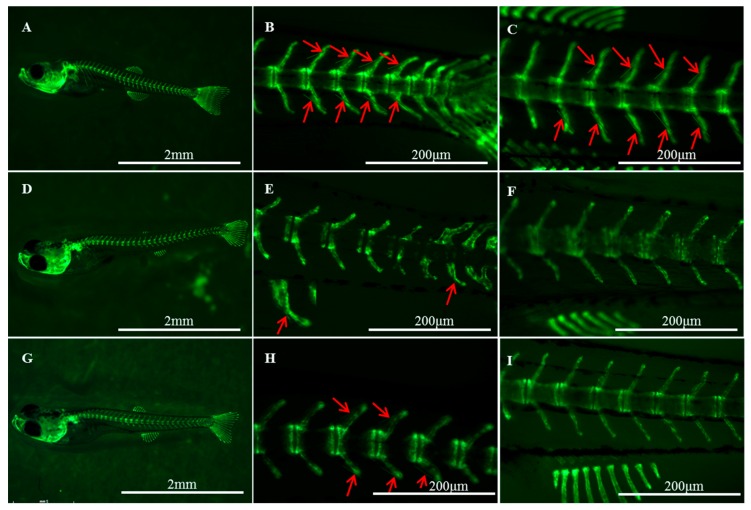
Effects of IWP-L6 (**D**–**F**) and K02288 (**G**–**I**) inhibitors on IB development in zebrafish larvae (Tg(sp7/GFP)). Control larvae (**A**–**C**) were treated with dimethyl sulfoxide (DMSO). Red arrows indicate IBs. (**B**,**E**,**H**) show the tail part; (**C**,**F**,**I**) show the dorsal part.

Two inhibitors, 15 μM K02288 and 5 μM IWP-L6, were used to treat zebrafish (Tg (sp7/eGFP)) larvae from 15 to 21 dph, respectively. A distinct decrease of sp7/eGFP expression was observed at 5 μM IWP-L6 upon exposure at 15 dph and part of the vertebra showed a curved phenomenon, whereas a slight decrease of sp7/eGFP expression was observed at 15 μM K02288 treatment (Supplementary Figure S8). IB development was significantly affected in the treatment groups (Figure 7A−C for the control group, Figure 7D−F for the IWP-L6-treated group, and Figure 7G−I for the K02288-treated group). At 25 dph, IBs were already ossified from tail to dorsal part in the control group; however, in the treatment groups, IBs were just beginning ossification in the tail part. The results indicated that both inhibitors significantly inhibited and delayed IB development.

## 3. Discussion

IBs, which are unique to teleost fishes, such as *M. amblycephala*, are formed in the myosepta and attach ligamentously to neural arches, centra and hemal arches [1,4]. To better understand IB ossified pattern, ARS was used to detect bone mineralization and Alcian blue staining was performed to visualize the cartilage [22,23,24]. ARS and alcian blue staining results from the four IB developmental stages indicated that IBs of *M. amblycephala* were membrane bone, which formed by undergoing intramembranous ossification without a cartilaginous phase. The previous studies had indicated that anteriorly oriented tendon in *Anchoa delicatissima*, the epaxial lateral tendons in *Rondeletia loricata* and *Afrocentric spinifer* were formed by endochondral ossification [1], which was different from the IB ossification pattern in the present study. Our histological results suggested that IB formation (at least for epineurals and epipleurals) is directly ossified from mesenchymal cells and then differentiate into osteoblasts [1,4,12].

Proteome at the different developmental stages of IB development of *M. amblycephala* showed that the phenotypic plasticity of proteome could partially account for regulation of IBs. Specifically, 3688 proteins were identified in IB developmental stages of *M. amblycephala* using iTRAQ MS/MS technology. There are limited studies about proteins related to fish bones. Proteins of extracellular matrix in zebrafish skeletal (including craniofacial, axial, and caudal fin) had been identified through using a MS-based approach [25]. The proteins of IBs and ribs from *M. amblycephala* had been comparatively analyzed by iTRAQ MS/MS [26]. Our present study may be considered as the first comparative and comprehensive proteomic study for IB development in fish species. The results from functional analysis with GO terms and KEGG pathways further demonstrated that the proteins identified in this study were associated with differentiation of osteoclasts and osteoblasts as well as bone formation, such as TGF-β signaling pathway, Wnt signaling pathway, osteoclast differentiation, MAPK signaling pathway, calcium signaling pathway, etc.

To identify bone-related proteins during IB development stages, the differentially expressed proteins of S2-vs-S1, S3-vs-S2, and S4-vs-S3 comparison groups were identified and functionally analyzed. At early stage of IB ossification, the proteins including thrombospondin-1, thrombospondin, and casq1a, showed up-regulated expression level. These four proteins may have a positive effect on differentiation of osteoblasts and IB formation. Thrombospondin and thrombospondin-1 had been found in multiple biological processes including angiogenesis, apoptosis, and activation of TGF-β [27], which are growth factor regulating osteoblasts. Calsequestrin is by far the most abundant Ca^2+^-binding protein in the sarcoplasmic reticulum of skeletal [28] and it also expressed in gilthead sea bream (*Sparus aurata*) vertebrae [29]. The up-regulation of casq1a may be important in regulating IB formation.

After S2, the IBs come into a rapid ossification and growth period. Parvalbumin and parvalbumin isoform 1c exhibited an increased expression level at S3. A previous study had reported that parvalbumin was a calcium-binding protein regulating Ca^2+^ [29]. Calcium existing in plasma could have an effect on bone mineralization [30]. Bone mineralization is an essential step during the development of bone and the previous study pointed out that IBs were ossified from tendons in the myosepta [1] and tendons mineralization is necessary for IB development. Tendon mineralization has long been recognized as a physiological adaptation found in some organisms, such as birds and dinosaurs [31], and calcium was crucial for tendon mineralization [32]. Therefore, the up-regulation of parvalbumin and parvalbumin isoform 1c indicated their positive effect on tendon mineralization during IB development.

Camk2a and transcription factor SOX-6 were identified and showed an up-regulated expression level at S3-vs-S2. Camk2a is a target protein of calmodulin which serves as a major Ca^2+^ sensor in eukaryotic cells and it expresses in osteoblasts [33,34]. Transcription factor SOX-6 acts to enhance functions, controlling both expression of extracellular genes and cell proliferation [35], and it had been defined in both mammals and *S. aurata* vertebrae [29]. Therefore, transcription factor SOX-6 and camk2a were up-regulated in S4-vs-S3 and may provide a positive signal for IB growth development.

To clearly clarify the expression of identified proteins at the transcript level, a combined analysis of proteins and mRNA during four key IB development stages was applied in this study and revealed a modest correlation between the two separate approaches, iTRAQ and RNAseq. To date, more and more correlation analyses of transcriptomic and proteomic have been used to analyze their biological variation. In fish species *A. japonica*, the hyperosmotic-responsive genes/proteins in gills were examined with the high-throughput transcriptomic and proteomic studies, which identified 51 hypo-responsive proteins [19]. In this study, the R value of correlation analysis between protein and mRNA in different comparison groups was not very high, especially S3-vs-S2, S4-vs-S2, and S4-vs-S3. It has been reported that mRNA abundance was shown to correlate poorly to the protein content [36,37,38], as it does not take into account the wide variety of post-translational modifications which are critical to protein functions [39,40].

To clearly elaborate the dynamics underlying the expression of bone-related proteins at the transcript level, 20 bone-regulated proteins were subjected to mRNA expression obtained by RNA-seq. Some proteins showed the same expression patterns at the mRNA level. At the early stage of IB formation, the five bone-regulated proteins (entpd5, pvalb, camk, collagen6α3, casq1a) showed an up-regulated expression at both levels. Entpd5 is specially expressed in osteoblasts and plays an essential role in bone mineralization [41]. Collagen 6α3 is an extracellular matrix protein that is the major component of skeletal muscles [42], and it was expressed in zebrafish skeletal extracellular matrix [25]. The other three proteins (casq1a, pvalb, camk) are related with Ca^2+^ and all have an effect on bone formation [43]. These five proteins being up-regulated at both levels in the present study, indicated an enhancement of osteoblast differentiation during IB ossification. Then, IBs came into a rapid growth stage from S2 to S3. Proteins that played a role in bone formation were also altered at S3-vs-S2, such as entpd5, anxa2a, anxa5, and ctsk. Anxa2a and anxa5 were thought as calcium-dependent phospholipid-binding proteins and involved in the calcium channel activity [44]. Moreover, a study reported that anxa 2a and 5 were associated with bone mineralization and highly expressed in osteoblasts [45]. Ctsk was involved in resorption of teleost bone and detected in *S. aurata* vertebrae, and then was associated with osteoclasts [46]. At S3-vs-S2, anxa1, anxa2a, anxa5, ctsk, and entpd5 were up-regulated at protein level but down-regulated at mRNA level, while pvalb and collagen6α3 were up-regulated at both levels in response to IB development, which potentially revealed the evidence that transcription and translation are differentially regulated and timed. All of the IBs in the tail part have basically appeared with a mature morphology and longer length at S4. The eight proteins (anxa5, nid1, camk, etc.) exhibited an up-regulated expression and may be involved in bone growth. Camk is a target protein of calmodulin and regulates osteoblast [34]. Nid1 was viewed as ECM and it was identified in zebrafish skeletal ECM [25]. At S4-vs-S3, camk, nid1, and anxa5 were up-regulated at protein level but down-regulated at mRNA level, while pvalb were up-regulated at both levels. These results will urge us to be careful when interpreting the mechanisms underlying protein expression at the transcriptional or translational level. The regulation roles of five bone-related genes (*casq1a*, *entpd5*, *camk*, *col6α3*, and *pvalb*) were validated at mRNA and protein levels. The protein expression has an increased trend from S1 to S4, whereas gene expression was up-regulated from S1 to S3. The two tenogenic genes (*scxb* and *tnmd*) also showed an increased expression from S1 to S3. Moreover, the expression of entpd5a and casq1a probes indicated the highest expression at S3 by in situ hybridization, which was consistent with mRNA expression. The results suggested that these genes have important regulation roles at transcription and translation levels during IB development.

Notably, the three proteins (collagen9α1, stat1, and tnc) associated with chondrocytes exhibited higher expression at S1, while had lower expression and their expression was not obviously changed at S2, S3, and S4. The stat1 is proposed to be one mediator of FGFR3 actions and regulates chondrocyte differentiation [47]. Tnc belongs to ECM but has an effect on chondrocyte differentiation [28]. The collagen9α1 is a part of cartilage structural proteins and acts on chondrogenesis and differentiation [29,48]. In this study, the three proteins had most abundant expression at S1 and exhibited lower expression at S2, S3, and S4, which further verified the point that IB formation (at least for epineurals and epipleurals) was not involved in chondrocyte at protein level.

To further certify the role of the bone-related pathway in bone formation, the two inhibitors (K02288 and IWP-L6) that were associated with TGF-β and the Wnt pathway were used to treat developing Tg (sp7/eGFP) zebrafish larva. The results indicated that the inhibitors significantly inhibited and delayed IB development, especially IWP-L6 inhibitor. Windhausen et al. [49] showed that the K02288 decreased bone mineralization on 2–3 days post-fertilization zebrafish cranial bone formation and showed a negative effect on osteoblast differentiation. The IWP-L6 was highly active in zebrafish and effectively inhibited posterior axis formation at low micromolar concentrations [50]. The inhibitors experiment in this study validated that TGF-β and Wnt pathways had an important role in IB development, which demonstrated that IBs follow the similar pathway regulatory mechanism as other bones in fish.

## 4. Methods

### 4.1. Methods

All experimental protocols in this study were approved by the Animal Experimental Ethical Inspection of Laboratory Animal Center, Huazhong Agricultural University, Wuhan, China (HZAUDO-2016-005, 2016-10-26). All efforts were made to minimize the suffering of the animals. All experiments were performed in accordance with relevant guidelines and regulations.

All fish used in this study were derived from offspring of *M. amblycephala* and incubated in the College of Fisheries of Huazhong Agricultural University. Tail muscles (manually remove skins, fins and vertebra) of four key stages (Figure 1A–D) were collected (100 fish at S1, 50 fish at S2, 20 fish at S3, 10 fish at S4, for each replicate) according to body length (detailed information was described according to Wan et al. [20]), and then snap-frozen in liquid nitrogen and stored at −80 °C for proteomics and gene expression analysis. To understand the histological differences in different stages, experimental fish (six fish for each stage) were maintained at 4% paraformaldehyde solution and then stained.

### 4.2. Fish Euthanasia

All experimental procedures involving fish were approved by the institution animal care and use committee of the Huazhong Agricultural University. Experimental fish were anaesthetized in well-aerated water containing the 100 mg/L concentration of Tricaine methanesulfonate (MS-222) before collecting sample.

### 4.3. Histological Analysis

After fixing the fish from four key IB developmental stages in 4% paraformaldehyde for 24 h, the tissues were dehydrated through a series of graded ethanol solutions (70–100%) and cleared in xylene at room temperature [51]. The tissues were then imbedded in paraffin at 60 °C. Transverse sections (5 μm thick) were prepared following the normal procedures [22,52]. Then the sections were stained withHE, alcian blue, and alizarin red S, respectively. Specifically, alcian blue is supposed to stain cartilaginous tissue [22,23], while bone stained by ARS is supposed to be associated with calcium deposition [22,53].

### 4.4. iTRAQ-Based Proteomics Analysis

#### 4.4.1. Protein Extraction and iTRAQ Labeling

Protein extraction and iTRAQ labeling were performed according to standard proteomics methods [54]. In brief, 100 μg of protein dissolved in iTRAQ (BGI, Shenzhen, China) dissolution buffer was subjected to reduction, alkylation, and digestion by trypsin, followed by desalting [55]. Regarding the four key stages of IB development, two biological replicates were prepared for each stage. Peptides derived from different samples were labeled with different isobaric tags.

#### 4.4.2. SCX Fractionation and LC-MS/MS Analysis

The dried iTRAQ-labeled peptides were prefractionated by offline Strong Cation Exchange (SCX) chromatography with a LC-20AB HPLC Pump system (Shimadzu, Japan). Reconstituted dried peptide fractions were analyzed using LC-MS/MS analysis based on Triple TOF 5600 (SCIEX, Framingham, MA, USA). The detailed information could be checked from our previous publication [26].

### 4.5. Protein Identification and Proteome Analyses

The obtained raw data files were converted into MGF files using Proteome Discoverer 1.2 (PD 1.2, Thermo, Shanghai, China), 5600 msconverter and the MGF file was searched. Mascot 2.3.02 (Matrix Science, London, UK) tool was used to identify proteins. For protein identification, a mass tolerance of 0.05 Da was permitted for intact peptide masses and 0.1 Da for fragmented ions, with allowance for one missed cleavage in the trypsin digests. Oxidation (M) and iTRAQ8plex (Y) were viewed as the potential variable modifications, and Carbamidomethyl (C), iTRAQ8plex (N-term), iTRAQ 8plex (K) as fixed modifications. The charge states of peptides were set to +2 and +3. Specifically, an automatic decoy database search was performed in Mascot by choosing the decoy check box in which a random sequence of database is generated and tested for raw spectra as well as the real database. To reduce the probability of false peptide identification, only peptides with significance scores (≥20) at the 99% confidence interval by a Mascot probability analysis greater than “identity” were counted as identified.

Functional annotations of the proteins were conducted using Blast2GO program against the non-redundant protein database (NR; NCBI; https://www.ncbi.nlm.nih.gov) and transcriptase data of intermuscular bones for four key IBs stages of *M. amblycephala* which had been established by our previous studies (SRR1613326). The KEGG database (http://www.genome.jp/kegg/) was used to classify and group these identified proteins.

### 4.6. Enrichment Analysis

For protein quantization, it was required that a protein contains at least two unique peptides, with a false discovery rate (FDR) < 0.01. The quantitative protein ratios were weighted and normalized by the median ratio in Mascot. Proteins with 1.5-fold change (up-regulated) or 0.5-fold change (down-regulated) and a *p*-value of statistical evaluation less than 0.05 in the comparison groups of S2-vs-S1, S3-vs-S2, S4-vs-S3, S3-vs-S1, S4-vs-S1, and S4-vs-S2 were determined as differentially abundant proteins.

### 4.7. Integrative Analysis of Proteomic and Transcriptomic Data

In our previous study, the transcriptomes databases from four key stages of IBs in *M. amblycephala* were sequenced and the corresponding unigenes were generated [20]. In this study, the obtained proteomics database was used to correlate the analysis with a transcriptomic database that had been online published. The R value was used to reveal linear correlation between proteomics and the transcriptomic database in each comparison group. The correlation analysis proteins were also annotated with GO and KEGG pathway (http://www.genome.jp/kegg/). The differentially expressed correlation proteins and mRNA abundance had a standard 1.5-fold change or 0.5-fold change and more than 0.8-gene significance.

### 4.8. Validation of Differentially Expressed Genes

Based on the transcriptomic and proteomic data, five genes (*entpd5*, *casq1a*, *pvalb*, *camk*, and col6α3) were associated with osteoblasts and bone formation and differentiation, as well as four tenogenic genes (*scxa*, *scxb*, *tnmd*, and *xirp2a*), were selected to validate from mRNA and protein levels. Quantitative real-time PCR (qRT-PCR) was performed according to SYBR Green Premix Ex Taq (TaKaRa, Da lian, China) using a QuantStudio™ 6 Flex real-time PCR System (Applied Biosystems, ABI, USA) according to the manufacturer’s instructions. Primers for each gene were designed using primer premier 5.0 based on the mRNA sequences obtained from the IB transcriptome database of *M. amblycephala* and shown in (Supplementary Table S6). The relative expression levels of the target genes were normalized to the housekeeping *M. amblycephala* gene β-actin, and further calculated using the double-standard curve method [56]. Western blot analysis was conducted by separating protein fragments using gel electrophoresis followed by transfer to a polyvinylidene difluoride membrane. The membrane was probed with primary mice entpd5 (1:500, bioss, Beijing, China), casq1a (1:1000, Abcam, Shanghai, China), pvalb (1:500, Abcam, Abcam, Shanghai, China), camk (1:1000, Abcam, Shanghai, China), col6α3 (1:2000, Abcam, Shanghai, China) antibody against GADPH (1:10000, Abcam, Shanghai, China), and stained with horseradish peroxidase (HRP)-conjugated secondary antibodies. Finally, band intensities were quantified using UN-SCAN-IT gel analysis software (version 6). The levels of protein expression were normalized to GADPH expression.

Two genes (*entpd5a* and *casq1a*) were selected to be validated by in situ hybridization method. The probes were made by in vitro transcription, followed by alkaline hydrolysis to yield fragments with a mean size of 800–900 nucleotides as assessed by polyacrylamide gel electrophoresis. Digoxigenin-labeled probes were made according to the manufacturer’s specifications (Roche; Mannheim, Germany) and were not subjected to alkaline hydrolysis because this treatment had no or few adverse effects on the sensitivity of hybridization. For sections of IBs key stages of *M. amblycephala*, paraffin-embedded tissues from six fish for each stage were sectioned with 5 μm then hybridized in situ, according to standard procedures [57].

### 4.9. Pathway Inhibitor Analysis

To assess the regulation role of TGF-β and Wnt signaling pathways during IB development process, we chose the fish model-zebrafish to conduct pathway inhibitor analysis. The zebrafish were raised according to standard protocols at Collage of Fisheries, Huazhong Agricultural University and zebrafish embryos and larval were cultured in fish water at 28 °C. The Tg (sp7/eGFP) transgenic line was obtained from Dr. Chung-Der Hsiao, Chung Yuan Christian University, Taiwan, and then was bred in College of Fisheries. The Tg (sp7/eGFP) transgenic line expresses osterix, which has conserved expression in osteoblasts [58]. The line was used to observe the vertebral and IB development.

The two inhibitors (K02288 and IWP-L6) were used to treat zebrafish larva. K02288 is a type of TGF-β/Smad inhibitor and presents a high specificity for BMP type I receptors [50], while IWP-L6 [50] inhibits Porcn, an essential regulator of Wnt ligand maturation and secretion [59,60]. Inhibitor stocks were diluted in dimethyl sulfoxide (DMSO) and then further diluted in fish water to give the required inhibitor concentrations. In order to find an appropriate concentration of inhibitor for treatment, the pre-experiment was conducted on 15 days post-hatch (dph) zebrafish with K02288 and IWP-L6 at 5, 15, and 25 μM for 48 h, respectively. Considering the mortality of fish and the effects of inhibition, the final treated concentrations of K02288 and IWP-L6 were set at 15 and 5 μM, respectively. Our previous study indicated that IBs were firstly ossified at the posterior part during 20–22 days post-hatch (dph) and then extended to the anterior parts in zebrafish [61]. Therefore, the inhibitors treatment was applied by immersing 15 dph Tg (sp7/eGFP) zebrafish larva in the fish water supplemented with DMSO as control group and the testing groups with chemical inhibitors (K02288 and IWP-L6). For each treatment, to minimize the amount of inhibitors used, experiments were performed in six-well plates with approximately five larvae per well in a volume of 5 mL. The fish were fed twice per day through transferring the fish from treatment solutions to the normal fish water for about 1 h. The treatments were continued for six days and the development of IBs was observed every day.

All images were taken on a M205 FA (Leica, Germany) stereomicroscope. Fluorescent images for Tg (sp7/eGFP) expression were obtained using GFPA filters (SZX16FL, ShanghaiChina.) For live imaging, larval were anaesthetized in MS-222 until they showed sufficiently low movement.

## 5. Conclusions

This study utilized iTRAQ methodology to construct the first proteomics map for four key stages of IB development in *M. amblycephala*. ARS and alcian blue staining results indicated that IB belongs to intramembranous ossification and did not involve the cartilaginous phase. The five proteins (entpd5, casq1a, pvalb, camk, and col6α3) associated with osteoblasts and bone formation and differentiation showed increasing expression trend from S1 to S4 and corresponded with the stained results with ARS staining. However, the three proteins (tnc, col9α1, and stat1) had the most abundant expression at S1 and exhibited lower expression at S2, S3, and S4, which verified that the IB formation was not involved in chondrocytes. We reported a combined analysis of the molecular response reflected by the transcriptome and proteome during four key stages of IB development in *M. amblycephala*. The results showed that the proteins were regulated by each other and displayed different expression trends in comparison groups. Moreover, the two inhibitors for TGF-β and Wnt pathways demonstrated their important role during IB development. Although additional work is required to evaluate the precise regulatory mechanisms of the identified proteins, integrated analysis of histological-transcriptomic-proteomic in the present study contributes a better understanding of the IB ossification process in teleosts.

## Figures and Tables

**Table 1:** Information of bone-related genes at protein and mRNA level.

NCBI nr Description	Abbreviation	S2-vs-S1		S3-vs-S2		S4-vs-S3	
		Protein	mRNA	Protein	mRNA	Protein	mRNA
Calsequestrin 1a precursor	casq1a	0.69	0.22	0.06	−0.13	−0.15	0.47
Calcium/calmodulin-dependent protein kinase type II subunit beta-like	camk	0.93	0.93	−0.17	0.77	0.30	−1.28
Parvalbumin	pvalb	0.68	1.27	1.32	0.73	0.11	1.66
Calreticulin precursor	calrl	−0.38	−1.71	−0.03	−4.08	−0.12	1.03
Annexin A1	anxa1	−0.12	0.09	0.21	−0.05	−0.43	0.43
Annexin A2a	anxa2a	−0.49	0.17	0.11	−0.13	−0.15	0.75
Annexin A5	anxa5	−0.40	−0.22	0.54	−0.88	0.08	−0.04
Ectonucleoside triphosphate diphosphohydrolase 5-like	entpd5	0.18	0.67	0.19	−0.18	−0.04	−0.23
Beta-catenin-like protein 1	β-catenin	−1.09	−0.49	−0.07	−0.42	0.11	−0.29
Transforming growth factor, beta-induced	TGF-β	−0.71	−0.02	−0.04	−1.25	−0.09	0.47
Tenascin C	tnc	−0.40	−1.08	−0.09	−1.48	0.06	0.25
Signal transducer and activator of transcription 1-alpha/beta	stat1	−0.97	0.24	−0.03	0.72	0.28	−1.12
Collagen alpha-1(IX) chain-like, partial	col9α1	−3.32	−9.38	0.73	0.00	−0.56	0.00
Laminin subunit beta-2 precursor	lamb2	−0.07	−0.42	0.01	−0.002	−0.64	−0.31
nidogen-1-like	nid1	0.11	−0.58	0.04	−0.67	0.15	−0.45
Collagen alpha-3(VI) chain-like	col6α3	0.57	1.67	0.56	0.06	−0.18	−1.12
Matrix metalloproteinase-14 precursor	mmp14b	0.44	−0.04	−0.17	−1.12	0.11	−0.40
Decorin variant 1	dcn	0.04	0.32	0.37	−1.60	−0.06	0.63
Cathepsin K	ctsk	−0.94	−0.22	0.82	−0.53	−0.79	−0.62
Prostaglandin E synthase 2-like	PGE2	−0.22	−0.40	0.12	−0.13	−0.20	−0.38

## Supplementary Materials

Supplementary materials can be found at https://www.mdpi.com/1422-0067/20/19/4698/s1.

## Abbreviations

DMSO	diluted in dimethyl sulfoxide;
dph	days post-hatch;
GO	gene ontology;
HE	hematoxylin-eosin;
IB	intermuscular bone;
ICAT	isotope-code affinity tags;
iTRAQ	Isobaric Tag for Relative Absolute Quantitation;
KEGG	Kyoto encyclopedia of genes and genomes;
mRNA	messenger RNA;
MS-222	Tricaine methanesulfonate;
qRT-PCR	Quantitative real-time polymerase chain reaction;
SILAC	stable isotopes labeling by amino acids in cell culture;
ARS	alizarin red S;
LC-MS/MS	liquid chromatography-tandem mass spectrometry.

## References

[1] Patterson C., Johnson G.D. (1995). The intermuscular bones and ligaments of Teleostean Fishes.

[2] Danos N., Ward A.B. (2012). The homology and origins of intermuscular bones in fishes: Phylogenetic or biomechanical determinants?. Biol. J. Linn. Soc. Lond..

[3] Nie C.H., Hilsdorf A.W.S., Wan S.M., Gao Z.X. (2019). Understanding the development of intermuscular bones in teleost: Status and future directions for aquaculture. Rev. Aquacult..

[4] Gemballa S., Britz R. (1998). Homology of intermuscular bones in Acanthomorph fishes. Am. Mus. Novit..

[5] Bing Z. (1962). On the myoseptal spines of the carp (*Cyprinus carpio* L.). Acta Zool. Sin..

[6] Bird N.C., Mabee P.M. (2003). Developmental morphology of the axial skeleton of the Zebrafish, *Danio rerio* (Ostariophysi: Cyprinidae). Dev. Dyn..

[7] Dong Z.J., Huang D.Z., Li L.J., Yuan X.H., Miao W.M., Chen Q.Q., Lu Z.B., Zhang B.L. (2006). Preliminary study on intermuscular bones of several cultured cyprinids. J Shanghai Fish Univ..

[8] Ke Z.H., Zhang W., Jiang Y., Bao B.L. (2008). Developmental morphology of the intermuscular bone in *Hypophthalmichthys molitrix*. Chin. J. Zool..

[9] Wan S.M., Yi S.K., Zhong J., Wang W.M., Jiang E.M., Chen B.X., Gao Z.X. (2014). Developmental and morphological observation of intermuscular bones in *Megalobrama amblycephala*. Acta Hydrobiol. Sin..

[10] Yao W.J., Gong X.L., Lü Y.P., Bao B.L. (2015). The ossificational process of the intermuscular bones in *Anguilla japonica*. J. Shanghai Ocean Univ..

[11] Hall B.K. (1988). The embryonic development of bone. Am. Sci..

[12] Karsenty G., Wagner E.F. (2002). Reaching agenetic and molecular understanding of skeletal development. Dev. Cell.

[13] Schaeffer B., Patterson C. (1984). Jurassic Fishes from the Western United States, with Comments on Jurassic Fish Distribution. Am. Mus. Novit..

[14] Ong S.E., Blagoev B., Kratchmarova I., Kristensen B., Steen H., Pandey A., Mann M. (2002). Stable isotope labeling by amino acids in cell culture, SILAC, as a simple and accurate approach to expression proteomics. Mol. Cell Proteom..

[15] Gygi S.P., Rist B., Gerber S.A., Turecek F., Gelb M.H., Aebersold R. (1999). Quantitative analysis of complex protein mixtures using isotope-coded affinity tags. Nat. Biotechnol..

[16] Bouchal P., Dvořáková M., Roumeliotis T., Bortlíček Z., Ihnatová I., Procházková I., Ho J.T., Maryáš J., Imrichová H., Budinská E. (2015). Combined Proteomics and Transcriptomics Identifies Carboxypeptidase B1 and Nuclear Factor κB (NF-κB) Associated Proteins as Putative Biomarkers of Metastasis in Low Grade Breast Cancer. Mol. Cell Proteom..

[17] Petersen H.O., Höger S.K., Mario L., Lengfeld T., Kuhn A., Warnken U., Nishimiya-Fujisawa C., Schnölzer M., Krüger M., Özbek S. (2015). A Comprehensive Transcriptomic and Proteomic Analysis of Hydra Head Regeneration. Mol. Biol. Evol..

[18] Long M., Zhao J., Li T., Tafalla C., Zhang Q., Wang X., Gong X., Shen Z., Li A. (2015). Transcriptomic and proteomic analyses of splenic immune mechanisms of rainbow trout (*Oncorhynchus mykiss*) infected by *Aeromonas salmonicida* subsp. salmonicida. J. Proteomics.

[19] Tse W.K., Sun J., Zhang H., Lai K.P., Gu J., Qiu J.W., Wong C.K. (2014). iTRAQ-based quantitative proteomic analysis reveals acute hypo-osmotic responsive proteins in the gills of the Japanese eel (*Anguilla japonica*). J. Proteom..

[20] Wan S.M., Yi S.K., Zhong J., Nie C.H., Guan N.N., Zhang W.Z., Gao Z.X. (2016). Dynamic mRNA and miRNA expression analysis in response to intermuscular bone development of blunt snout bream (*Megalobrama amblycephala*). Sci. Rep..

[21] Muers M. (2011). Gene expression: Transcriptome to proteome and back to genome. Nat. Rev. Genet..

[22] Eames B.F., Yan Y.L., Swartz M.E., Levic D.S., Knapik E.W., Postlethwait J.H., Kimmel C.B. (2011). Mutations in fam20b and xylosyltransferase1 reveal that cartilage matrix controls timing of endochondral ossification through inhibition of chondrocyte maturation. PLoS Genet..

[23] Walker M.B., Kimmel C.B. (2007). A two-color acid-free cartilage and bone stain for zebrafish larvae. Biotech. Histochem..

[24] Witten P.E., Huysseune A. (2009). A comparative view on mechanisms and functions of skeletal remodelling in teleost fish, with special emphasis on osteoclasts and their function. Biol. Rev. Camb. Philos. Soc..

[25] Kessels M.Y., Huitema L.F., Boeren S., Kranenbarg S., Schulte-Merker S., van Leeuwen J.L., de Vries S.C. (2014). Proteomics analysis of the zebrafish skeletal extracellular matrix. PLoS ONE.

[26] Nie C.H., Wan S.M., Tomljanovic T., Treer T., Hsiao C.D., Wang W.M., Gao Z.X. (2017). Comparative proteomics analysis of teleost intermuscular bones and ribs provides insight into their development. BMC Genom..

[27] Amend S.R., Uluckan O., Hurchla M., Leib D., Novack D.V., Silva M., Frazier W., Weilbaecher K.N. (2015). Thrombospondin-1 regulates bone homeostasis through effects on bone matrix integrity and nitric oxide signaling in osteoclasts. J. Bone Miner. Res..

[28] Beard N.A., Laver D.R., Dulhunty A.F. (2004). Calsequestrin and the calcium release channel of skeletal and cardiac muscle. Prog. Biophys. Mol. Biol..

[29] Vieira F.A., Thorne M.A., Stueber K., Darias M., Reinhardt R., Clark M.S., Gisbert E., Power D.M. (2013). Comparative analysis of a teleost skeleton transcriptome provides insight into its regulation. Gen. Comp. Endocrinol..

[30] Talmage R.V., Matthews J.L., Mobley H.T., Lester G.E. (2003). Calcium homeostasis and bone surface proteins, a postulated vital process for plasma calcium control. J. Musculoskelet Neuronal Interact.

[31] Vandenberge J.C., Storer R.W. (1995). Intratendinous Ossification in Birds-a Review. J. Morphol..

[32] Qu J., Thoreson A.R., Chen Q., An K.N., Amadio P.C., Zhao C. (2013). Tendon gradient mineralization for tendon to bone interface integration. J. Orthop. Res..

[33] Gifford J.L., Ishida H., Vogel H.J. (2012). Structural Characterization of the Interaction of Human Lactoferrin with Calmodulin. PLoS ONE.

[34] Doroudi M., Plaisance M.C., Boyan B.D., Schwartz Z. (2015). Membrane actions of 1α, 25(OH)2D3 are mediated by Ca(2+)/calmodulin-dependent protein kinase II in bone and cartilage cells. J. Steroid Biochem. Mol. Biol..

[35] Smits P., Li P., Mandel J., Zhang Z., Deng J.M., Behringer R.R., de Crombrugghe B., Lefebvre V. (2001). The transcription factors L-Sox5 and Sox6 are essential for cartilage formation. Dev. Cell.

[36] Núñez C., Esteve-Núñez A., Giometti C., Tollaksen S., Khare T., Lin W., Lovley D.R., Methé B.A. (2006). DNA microarray and proteomic analyses of the RpoS regulon in Geobacter sulfurreducens. J. Bacteriol..

[37] Anderson L., Seilhamer J. (1997). A comparison of selected mRNA and protein abundances in human liver. Electrophoresis.

[38] Hegde P.S., White I.R., Debouck C. (2003). Interplay of transcriptomics and proteomics. Curr. Opin. Biotechnol..

[39] Nie L., Wu G., Culley D.E., Scholten J.C., Zhang W. (2007). Integrative analysis of transcriptomic and proteomic data: Challenges, solutions and applications. Crit. Rev. Biotechnol..

[40] Karve T.M., Cheema A.K. (2011). Small changes huge impact: The role of protein posttranslational modifications in cellular homeostasis and disease. J. Amino Acids.

[41] Huitema L.F., Apschner A., Logister I., Spoorendonk K.M., Bussmann J., Hammond C.L., Schulte-Merker S. (2012). Entpd5 is essential for skeletal mineralization and regulates phosphate homeostasis in zebrafish. Proc. Natl. Acad. Sci. USA.

[42] Irwin W.A., Bergamin N., Sabatelli P., Reggiani C., Megighian A., Merlini L., Braghetta P., Columbaro M., Volpin D., Bressan G.M. (2003). Mitochondrial dysfunction and apoptosis in myopathic mice with collagen VI deficiency. Nat. Genet..

[43] Fonseca V.G., Rosa J., Laizé V., Gavaia P.J., Cancela M.L. (2011). Identification of a new cartilage-specific S100-like protein up-regulated during endo/perichondral mineralization in gilthead seabream. Gene Expr. Patterns.

[44] Moss S.E., Morgan R.O. (2004). The annexins. Genome Biol..

[45] Kirsch T. (2005). Annexins-their role in cartilage mineralization. Front Biosci..

[46] Azuma K., Kobayashi M., Nakamura M., Suzuki N., Yashima S., Iwamuro S., Ikegame M., Yamamoto T., Hattori A. (2007). Two osteoclastic markers expressed in multinucleate osteoclasts of goldfish scales. Biochem. Biophys. Res. Commun..

[47] Sahni M., Ambrosetti D.C., Mansukhani A., Gertner R., Levy D., Basilico C. (1999). FGF signaling inhibits chondrocyte proliferation and regulates bone development through the STAT-1 pathway. Genes Dev..

[48] Sophia Fox A.J., Bedi A., Rodeo S.A. (2009). The basic science of articular cartilage: Structure, composition, and function. Sports Health.

[49] Windhausen T., Squifflet S., Renn J., Muller M. (2015). BMP Signaling Regulates Bone Morphogenesis in Zebrafish through Promoting Osteoblast Function as Assessed by Their Nitric Oxide Production. Molecules.

[50] Wang X., Moon J., Dodgem M.E., Pan X., Zhang L., Hanson J.M., Tuladhar R., Ma Z., Shi H., Williams N.S. (2013). The development of highly potent inhibitors for porcupine. J. Med. Chem..

[51] Xiong S., Wu J., Jing J., Huang P.P., Li Z., Mei J., Gui J.F. (2017). Loss of stat3 function leads to spine malformation and immune disorder in zebrafish. Sci. Bull..

[52] Luzio A., Monteiro S.M., Rocha E., Fontaínhas-Fernandes A.A., Coimbra A.M. (2016). Development and recovery of histopathological alterations in the gonads of zebrafish (*Danio rerio*) after single and combined exposure to endocrine disruptors (17α-ethinylestradiol and fadrozole). Aquat. Toxicol..

[53] Matsuoka F., Takeuchi I., Agata H., Kagami H., Shiono H., Kiyota Y., Honda H., Kato R. (2013). Morphology-based prediction of osteogenic differentiation potential of human mesenchymal stem cells. PLoS ONE.

[54] Han Z., Sun J., Zhang Y., He F., Xu Y., Matsumura K., He L.S., Qiu J.W., Qi S.H., Qian P.Y. (2013). iTRAQ-based proteomic profiling of the barnacle Balanus amphitrite in response to the antifouling compound meleagrin. J. Proteome Res..

[55] Dineshram R., Sharma R., Chandramouli K., Yalamanchili H.K., Chu I., Thiyagarajan V. (2015). Comparative and quantitative proteomics reveal the adaptive strategies of oyster larvae to ocean acidification. Proteomics.

[56] Larionov A., Krause A., Miller W. (2005). A standard curve based method for relative real time PCR data processing. BMC Bioinf..

[57] Moorman A.F., Houweling A.C., de Boer P.A., Christoffels V.M. (2001). Sensitive nonradioactive detection of mRNA in tissue sections: Novel application of the whole-mount in situ hybridization protocol. J. Histochem. Cytochem..

[58] Kague E., Roy P., Asselin G., Hu G., Simonet J., Stanley A., Albertson C., Fisher S. (2016). Osterix/Sp7 limits cranial bone initiation sites and is required for formation of sutures. Dev. Biol..

[59] Kadowaki T., Wilder E., Klingensmith J., Zachary K., Perrimon N. (1996). The segment polarity gene porcupine encodes a putative multitransmembrane protein involved in Wingless processing. Genes Dev..

[60] Komekado H., Yamamoto H., Chiba T., Kikuchi A. (2007). Glycosylation and palmitoylation of Wnt-3a are coupled to produce an active form of Wnt-3a. Genes Cells.

[61] Nie C.H., Chen Z.X., Dai C.J., Wan S.H., Gao Z.X. (2018). Ossification patterns of intermusclar bones in different fish species. Acta Hydrobiol. Sin..

