# Supplementary material for: Development of Teleost Intermuscular Bones Undergoing Intramembranous Ossification Based on Histological-Transcriptomic-Proteomic Data
Source: Int J Mol Sci. 2019 Sep 22;20(19):4698. doi: 10.3390/ijms20194698 (PMC6801895; doi:10.3390/ijms20194698)
Supplement: Supplementary file 1 [file ijms-20-04698-s001.zip › ijms-581572 supplementary/Supplementary Files/Supplement figures.pdf]

Development of teleost intermuscular bones undergoing intramembranous ossification based on histological-transcriptomic-proteomic data

Chun-Hong Nie [1,2], Shi-Ming Wan [1,2], Yu-Long Liu [4], Han Liu [1,2], Wei-Min Wang [1,2] and Ze-Xia Gao [1,2,3*]

[1] College of Fisheries, Key Lab of Agricultural Animal Genetics, Breeding and Reproduction of Ministry of Education/Key Lab of Freshwater Animal Breeding, Ministry of Agriculture, Huazhong Agricultural University, Wuhan, Hubei 430070, China; 1508085740@qq.com (C.H. N); 276222007@qq.com (S.M. W); 592299190@qq.com (H. L); wangwm5911@gmail.com (W.M. W)

[2] Engineering Technology Research Center for Fish Breeding and Culture in Hubei Province, Wuhan 430070, China

[3] National Demonstration Center for Experimental Aquaculture Education, Huazhong Agricultural University, Wuhan 430070, China

[4] Department of Molecular and Cellular Biology, University of California Davis, Davis 95616, California, USA; idrliu@ucdavis.edu (Y.L. L)

* Correspondence: gaozexia@hotmail.com; Tel/Fax: +86-27-8728-2113

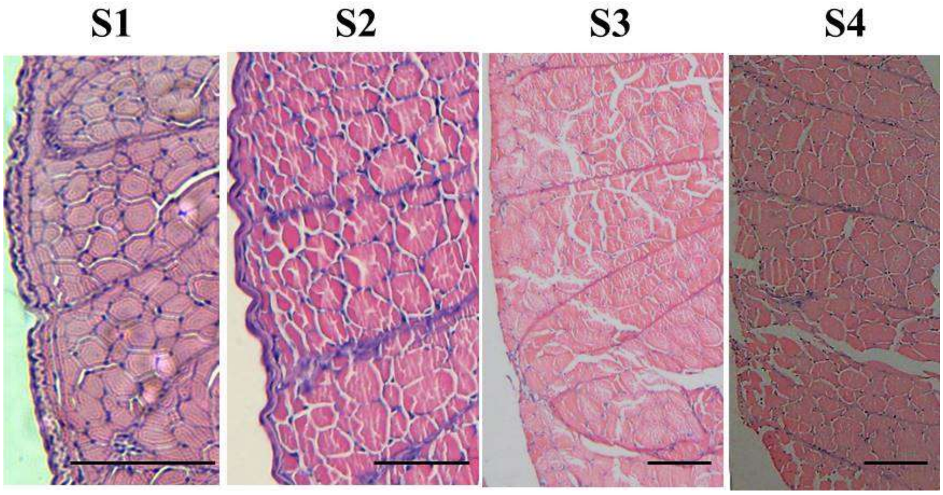

Figure S1 The histology structure with HE staining of muscle tissues at four stages. Scales bars = 50 μm.

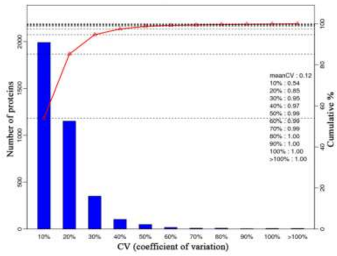

S2-vs-S1

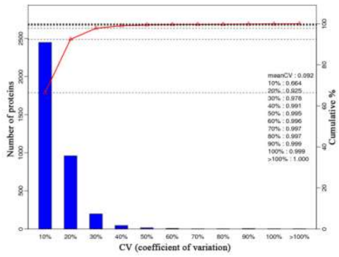

S3-vs-S2

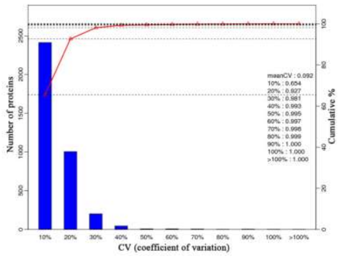

S4-vs-S3

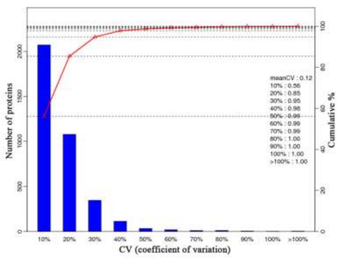

S3-vs-S1

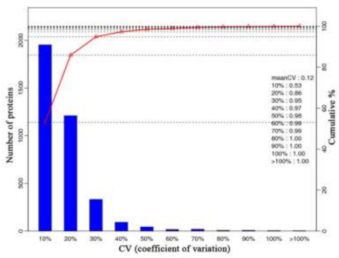

S4-vs-S2

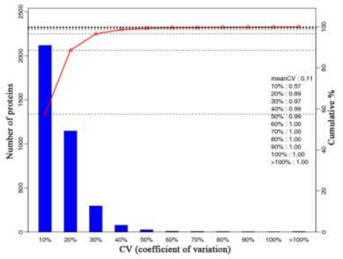

S4-vs-S2

Figure S2 Repeatability analysis of data obtained from iTRAQ in different groups.

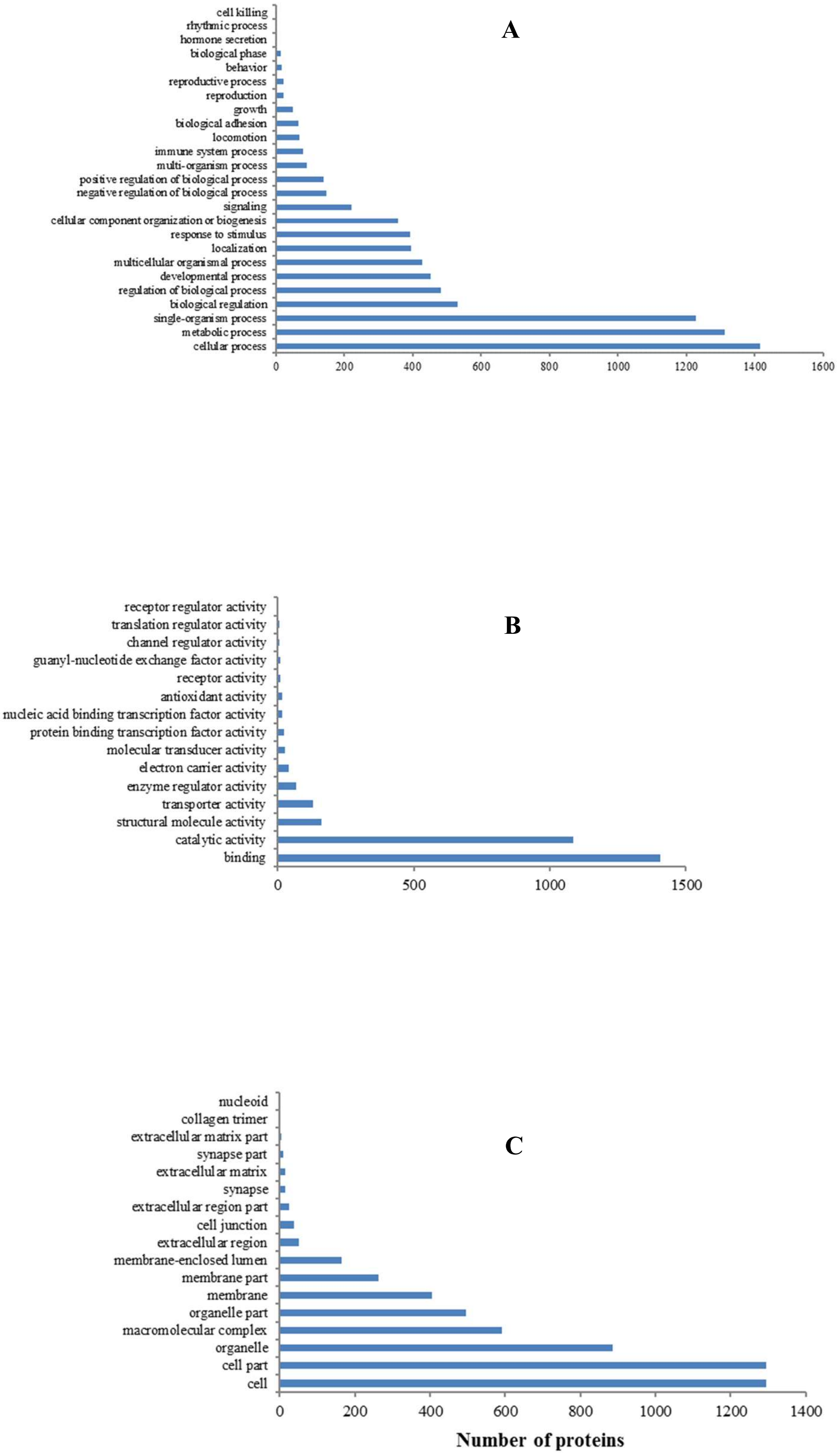
A
cell killing
rhythmic process
hormone secretion
biological phase
behavior
reproductive process
reproduction
growth
biological adhesion
locomotion
immune system process
multi-organism process
positive regulation of biological process
negative regulation of biological process
signaling
cellular component organization or biogenesis
response to stimulus
localization
multicellular organismal process
developmental process
regulation of biological process
biological regulation
single-organism process
metabolic process
cellular process
0
200
400
600
800
1000
1200
1400
1600
B
receptor regulator activity
translation regulator activity
channel regulator activity
guanyl-nucleotide exchange factor activity
receptor activity
antioxidant activity
nucleic acid binding transcription factor activity
protein binding transcription factor activity
molecular transducer activity
electron carrier activity
enzyme regulator activity
transporter activity
structural molecule activity
catalytic activity
binding
0
500
1000
1500
C
nucleoid
collagen trimer
extracellular matrix part
synapse part
extracellular matrix
synapse
extracellular region part
cell junction
extracellular region
membrane-enclosed lumen
membrane part
membrane
organelle part
macromolecular complex
organelle
cell part
cell
0
200
400
600
800
1000
1200
1400
Number of proteins

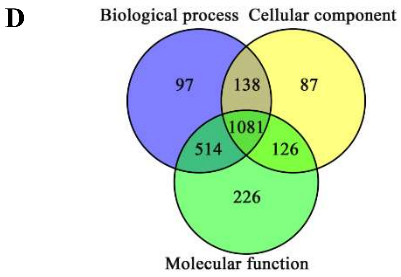

Figure S3 Proteins were functionally annotated for (**A**) biological process, (**B**) molecular function, as well as (**C**) cellular component and Venn diagram of proteins annotated for three processes (**D**).

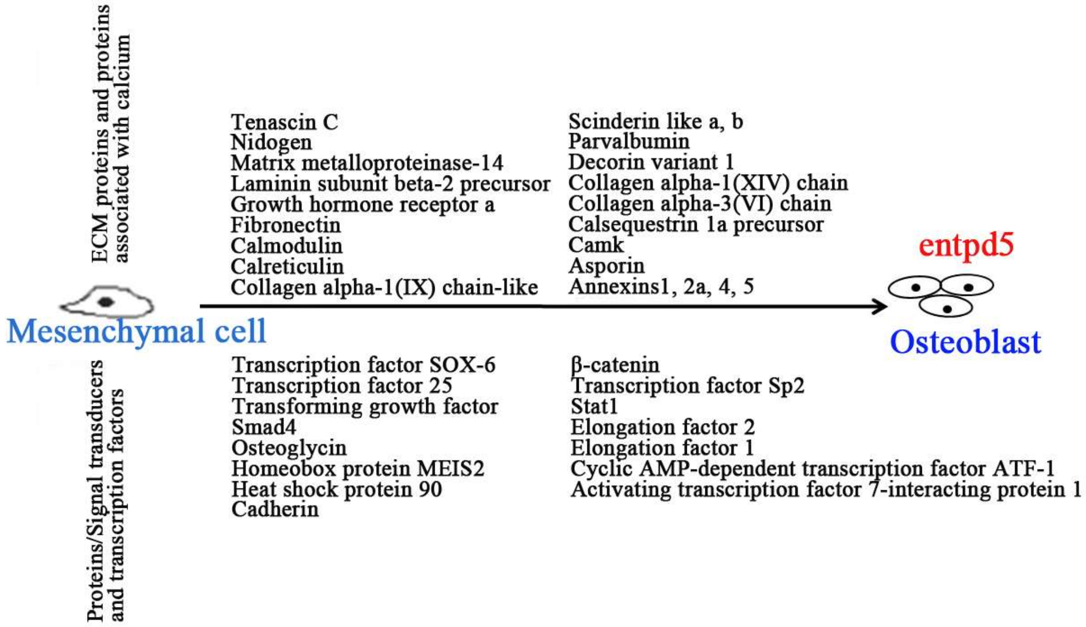

Figure S4 Schematic representation of the key proteins in osteoblastogenesis during IB ossification process.

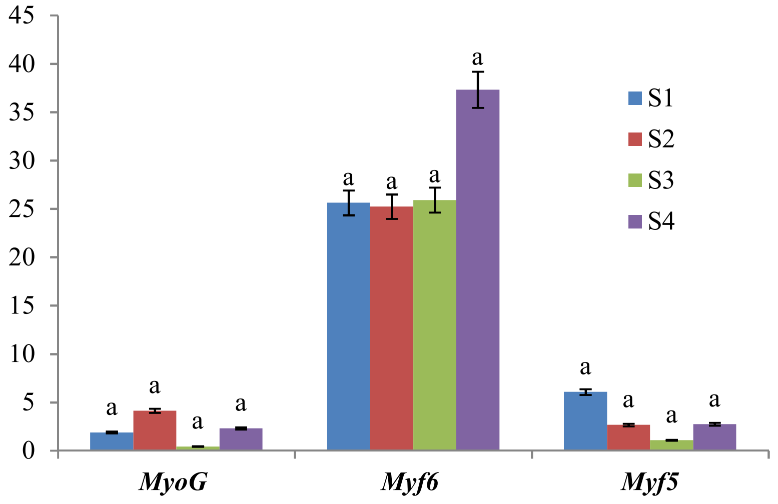

Figure S5 Expression of muscle-related genes at different stages based on RNA-seq data.

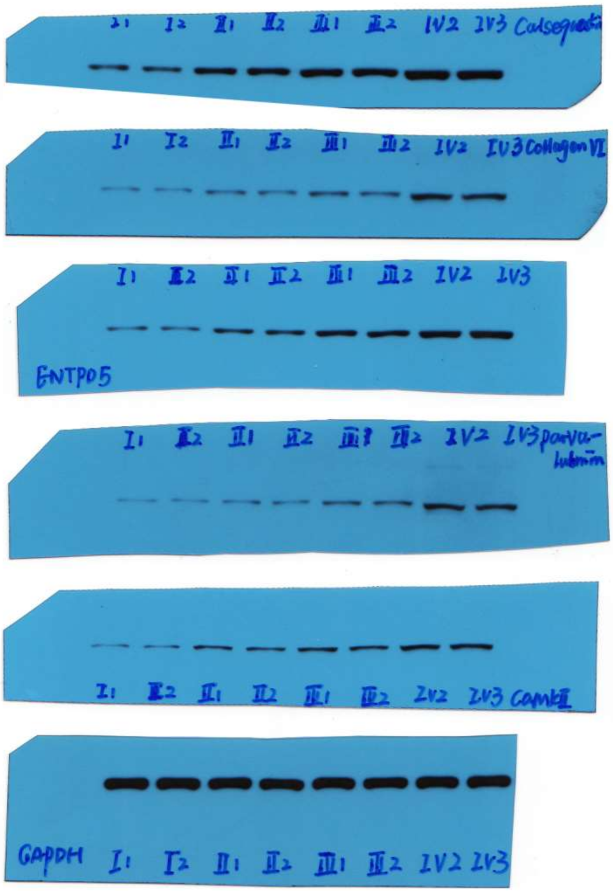

Figure S6 The raw data photo of western blot for five proteins; GAPDH is used as a loading control.

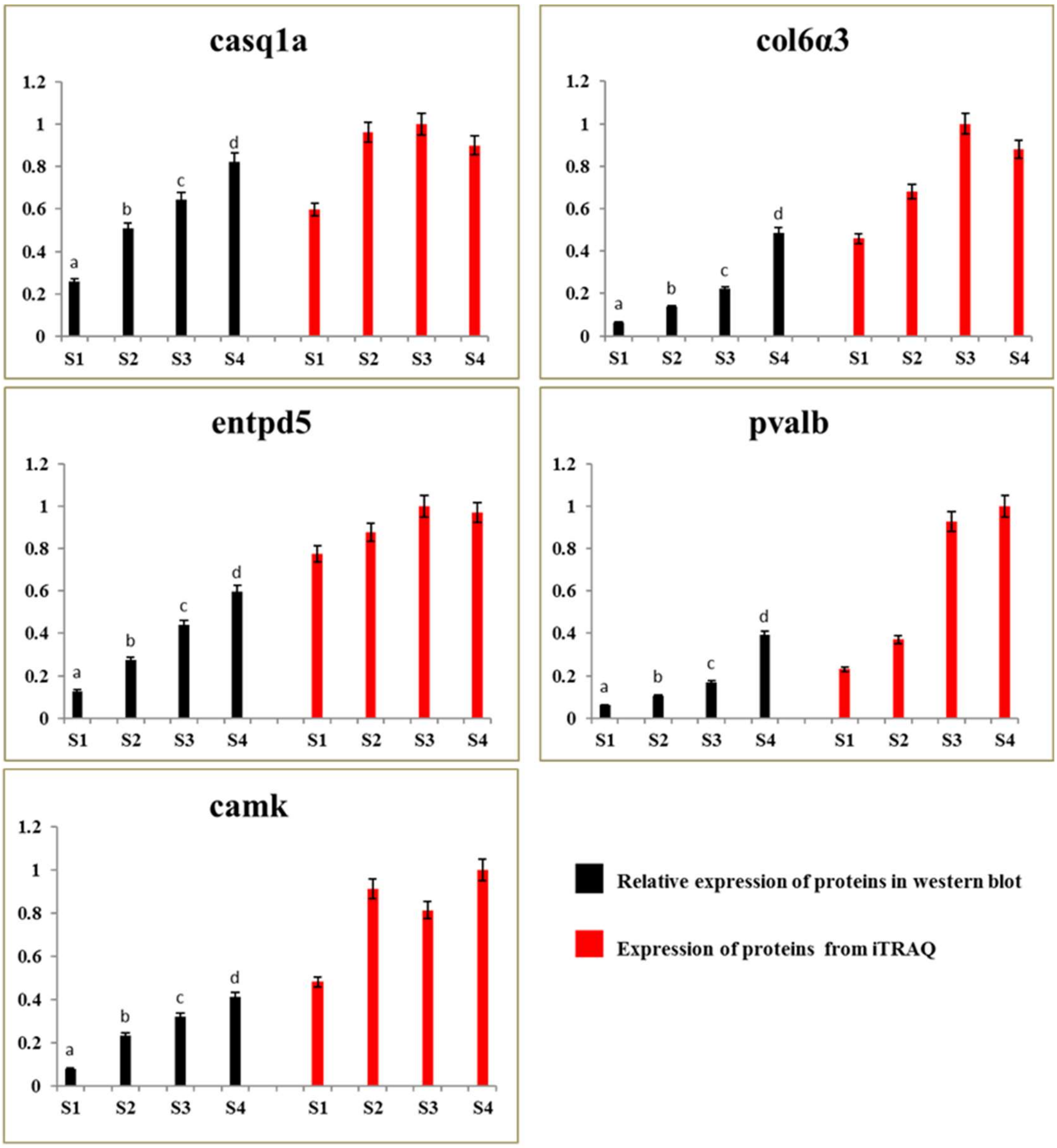

Figure S7 Comparison of expression levels for the 5 detected bone-related proteins based on iTRAQ and western blot results. Different letters above pillars for each stage indicated statistical significance at the level of $p < 0.05$.

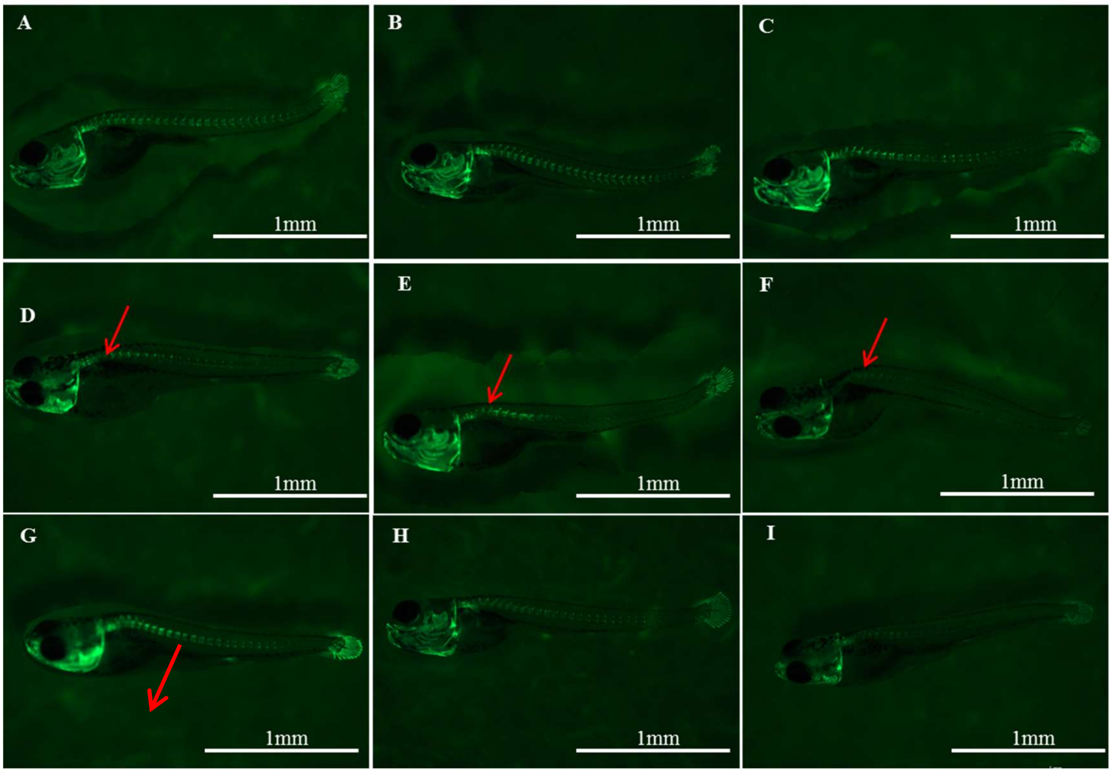

Figure S8 Effects of IWP-L6 (D–F) and K02288 (G–I) on Tg (*sp7/eGFP*) expression of vertebrate in zebrafish larvae. Control larvae (A, B, C) were treated with DMSO. Red arrows represent that part of vertebrate had a curved phenomenon.
